# Ultimate Functional Preservation With Intersphincteric Resection for Rectal Cancer

**DOI:** 10.3389/fonc.2020.00297

**Published:** 2020-03-05

**Authors:** Maxime Collard, Jérémie H. Lefevre

**Affiliations:** Sorbonne Université, Department of Digestive Surgery, AP-HP, Hôpital Saint Antoine, Paris, France

**Keywords:** rectal cancer, intersphincteric resection (ISR), functional results, neoadjucant chemoradiation, LARS—low anterior resection syndrome

## Abstract

The proximity of the very low rectum rectal cancer to the anal sphincter raises a specific problem: how and until when can we preserve the anal continence without compromising the oncological result of the tumor resection? In this situation, intersphincteric resection (ISR) offers an excellent alternative to abdominoperineal resection (APR), but the selection of patients for this option must be extremely precise. This complex choice justifies the simultaneous consideration of an oncological approach with a functional approach in order to provide a full benefit to the patient. When a circumferential resection margin of at least 1 mm can be performed with a distal resection margin of at least 1 cm with or without preoperative radiotherapy, ISR ensures a safety choice. The oncological results of ISR reported in the literature when performed properly found a 5-year disease-free survival of 80.2% with a local recurrence rate of only 5.8%. In parallel to this oncological evaluation, the expected post-operative functional outcome and the resulting quality of life must be properly assessed pre-operatively, since partial or total resection of the internal sphincter impacts significantly on the functional outcome. Based on data from the literature, this work reports the essential anatomical considerations and then the oncological and functional elements indispensables when an anal continence preservation is evoked for a tumor of the very low rectum. Finally, the precise selection criteria and the major surgical principles are outlined in order to guarantee the safety of this modern choice for the patient.

## Introduction

The quality of rectal cancer surgery has improved considerably in recent years as a result of the emergence of key concepts such as the total mesorectal excision proposed by Heald et al. (1). These advanced have made it possible to standardize the surgical technique with the dual objective of guaranteeing both the best oncological result and the best functional result without a permanent stoma. This dual objective is particularly difficult for tumors of the very low rectum due to their proximity to the anal sphincter. The radical solution by abdomino-perineal resection (APR) was the historical choice option for these tumors, in particular to ensure the distal and lateral safety margin (2). However, the evolution of knowledge on minimum margins of safety and the discovery of intersphincteric resection (ISR) (3) have considerably reduced the place of APR in patients with very low rectal cancer (4).

However, ISR indications are not a clear-cut issue. An overly extreme attitude toward ISR indications is dangerous because it may affect the oncological survival of patients on the one hand and lead to an unacceptable functional result on the other. The effectiveness of neoadjuvant radiochemotherapy makes patient selection even more complex. The objective of this work is therefore to take stock of the right indications of ISR in view of the data in the literature on the subject.

## Intersphincteric Resection: Anatomical Considerations

**Figure 1:**
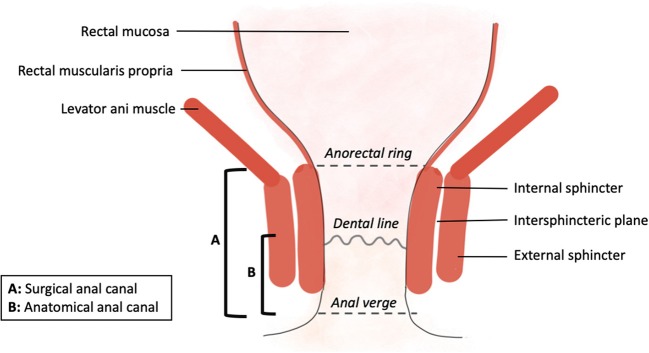
Anatomy of the very low rectum.

**Figure 2:**
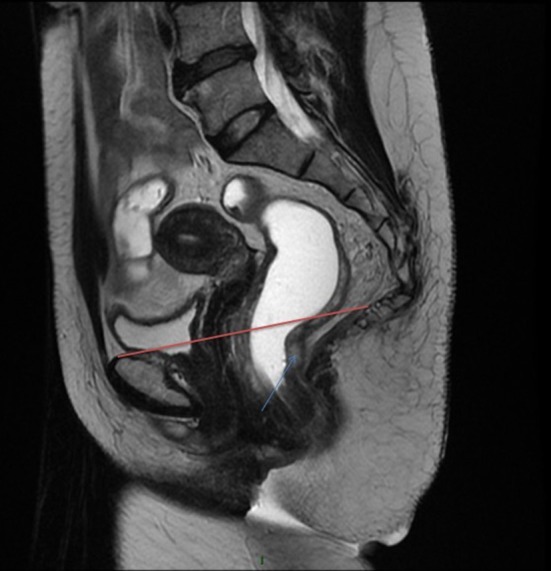
Definition of the low rectum on MRI. On this sagittal view in a MRI T2 sequence, the low rectum begins below the red line the origin of the levator muscles and the pubic bone.

Anatomical knowledge of the lower rectum and anal canal is an essential prerequisite for understanding the ISR problem (Figure 1). From a synthetic and practical point of view, the important anatomical points are:

- The low rectum is usually defined as the lower third of the rectum within 5–6 cm from the anal verge (5) or 2 cm above the dentate line. This distal part of the rectum can be identified as the rectal zone below the origin of the levator ani muscle where the mesorectum fuses with the rectosacral fascia and tapers at the anorectal ring. Other authors have defined the low rectum on MRI (magnetic resonance imaging) including all tumors with a lower tumoral edge below a line between the origin of the levator muscles and the pubic bone [(6); Figure 2].
- The anorectal ring corresponds to the U-shaped sling of striated muscle from the puborectalis muscle that pulls anteriorly the rectum and delimits the beginning of the surgical anal canal.
- The surgical anal canal extends from the anorectal ring to the anal verge and includes the external and internal sphincters. The anatomical anal canal is the distal part of the surgical anal canal and extends from the dental line to the anal verge. Histologically, it corresponds to the transitional epithelium.
- The length of the anal canal varies considerably from one individual to another. On average, the length of the surgical anal canal was 4.2 cm and that of the anatomical anal canal was 2.1 cm in a study dedicated to this question (7).
- The internal anal sphincter is formed by a very thickened segment of the circular muscle layer of the distal rectum. The external sphincter consists of skeletal muscle fibers that mix with the puborectal component of the levator ani to form a muscle ring. The space between internal and external sphincter constitutes the intersphincteric plane.

**Figure 3:**
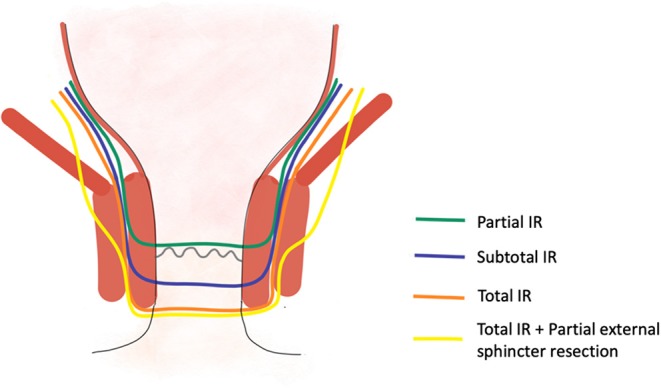
Classification of the very low rectal cancer according to the Rullier's classification (8). The dotted lines indicate the surgical dissection plan required based on the location of the lesion.

It is crucial to obtain a precise localization of the tumor in the lower rectum to be able to evaluate the appropriate surgical intervention to be proposed. In order to homogenize the discussion on tumors of the lower rectum, Rullier et al. proposed a four-stage classification [(8); Figure 3]. Type I low rectal cancers are supra-anal tumors, that is, lesions >1 cm from the anorectal ring. Type II are juxta-anal tumors, that is, lesions ≤ 1 cm from the anorectal ring. Type III are intra-anal tumors, that is, lesions with internal anal sphincter invasion. Type IV are transanal tumors, that is, lesions with external anal sphincter or levator ani muscle invasion.

It is from the knowledge of these anatomical reminders and the classification of Rullier et al. that the place of ISR can be correctly investigated. In practice, ISR concerns stage II and III lesions. Some authors have assessed for stage IV tumors to propose ISR combined with partial resection of the external sphincter (9, 10).

**Figure 4:**
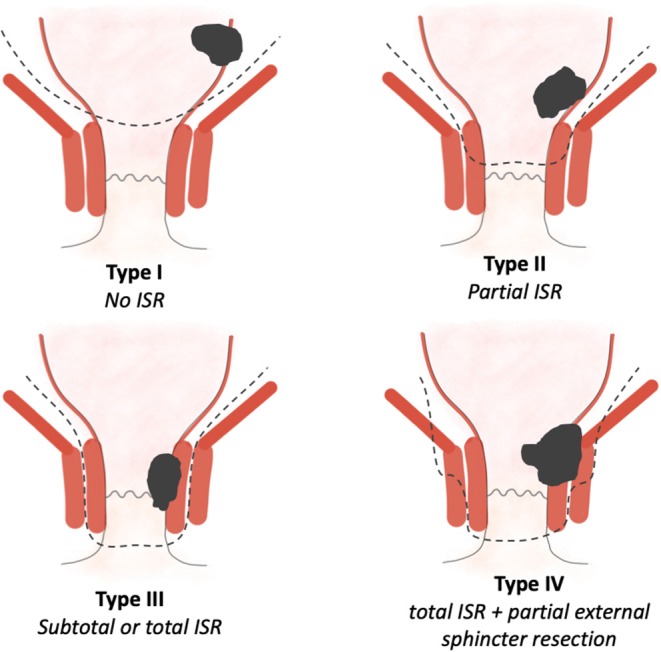
Four different options of intersphincteric resection.

In total, there are four types of ISR for lower rectal lesions stage II/III and some stage IV lesions that have been proposed in the literature [(9); Figure 4]:

- Partial ISR: Incision at the level of the dentate line or just below, removing one-third or half of the internal sphincter.
- Subtotal ISR: Incision 1–2 cm below the dentate line, removing two third of the internal sphincter.
- Total ISR: Incision 2 cm below the dentate line, removing the entire internal sphincter.
- Total ISR + Partial external sphincter resection: Incision 2 cm below the dentate line, removing the entire internal sphincter and removing partially the external sphincter.

The clinical use of these theoretical knowledges requires a correct classification of the lesion when ISR is being considered. It is therefore necessary to combine the examinations and converge the results to propose a precise classification of the tumor. Data from digital rectal examination, endoluminal ultrasound, and MRI are required to provide an efficient local staging (11). MRI is particularly effective in assessing the localization of the tumor, the invasion of internal and external sphincter and other adjacent structures such as the anus elevating muscle (12).

The rise of neoadjuvant treatments, particularly radiochemotherapy, makes the pre-operative assessment of lower rectal lesions even more complex. Indeed, it is particularly difficult to evaluate the tumor response on imaging and to differentiate fibrosis and persistent tumor. A recent meta-analysis reports a T-stage restaging accuracy of 52% for MRI and 65% for endoluminal ultrasound without significant difference between these two imaging modalities (13). In addition, this work highlights the high heterogeneity of studies on this subject, probably related to several factors such as variation in the delay between the radiotherapy and the imaging restaging, heterogeneity of imaging modalities (type of MRI sequence, 1.5 or 3 Tesla…) and finally a variable experience of the radiologist to interpret these complex exams (14).

Adding functional sequences to the MRI, could improve the performance in this regard. The perfusion sequences have been the subject of encouraging preliminary works, showing a satisfactory correlation between tumor response and tumor perfusion parameters (15), but this work has yet to be confirmed in larger studies. The diffusion sequences also make it possible to raise sensitivity to the evaluation of tumor response. It has been shown that the apparent diffusion coefficient was higher in tumors in response to radiochemotherapy than in non-replying tumors (16). It would increase the distinction between tumor fibrosis and residual tumor. However, these data are very preliminary and reported only in small series.

All of these data underline that re-evaluation of tumor after radiochemotherapy, particularly in order to propose bowel continuity preservation by ISR that was not possible before neoadjuvant treatment, is a particularly challenging situation and requires a multidisciplinary discussion, including specialized radiologists.

## Oncological Approach

Oncological results of ISR reported in the literature found a 5-year disease-free survival of 80.2% with a local recurrence rate of 5.8% (Table 1). More details about oncological results after ISR from different teams are presented in Table 1. The oncological challenge of ISR is to ensure safe circumferential and distal resection margins (CRM and DRM).

**Table 1:** Oncological outcomes after intersphincteric resection from studies including at least 100 patients.

**References**	***n***	**Median follow-up (months)**	**R0 resectio*n* (%)**	**Local recurrence (%)**	**Distant recurrence (%)**	**5-year overall survival (%)**	**5-year disease-free survival (%)**
Bannon et al. (17)	109	40	–	6.7	11	91	77
Schiessel et al. (18)	121	94	96.7	5.3	–	88	–
Saito et al. (9)	225	41	98.7	3.6	9	92	83
Portier et al. (19)	173	67	–	8.6	17.6	86.1	83.9
Akasu et al. (20)	120	42	96.7	6.7	13	91	77
Saito et al. (21)	132	40	100	10.6	24	80	69
Laurent et al. (22)	175	53	88	3.5	22.4	90	84
Akagi et al. (23)	124	65	–	4.8	10.5	–	–
Lee et al. (24)	163	53	–	11.0	20.2	–	–
Tsukamoto et al. (25)	112	60	92.9	–	–	–	73.3
Rouanet et al. (26)	400	49	96	–	–	–	–
Yamada et al. (27)	107	41	100	2.5	20.5	92	87
Parks et al. (28)	147	34	91.4	11.7	22.4	–	–
Kim et al. (29)	488	–	98	2.5	15.8	86.7	80.7
Weighted mean	2,596	52	96	5.8	16.7	88.2	80.2

The CRM to be obtained is similar to that of standard proctectomy, i.e., CRM > 1 mm in order to avoid the risk of local recurrence (30) and of distant metastases (31). The specificity of ISR is the absence of mesorectum around the very low rectum. It is therefore important to be assured that the intersphincteric plane is not invaded by the tumor before proposing ISR with preservation of the external sphincter.

The DRM has its own specificities for patients eligible for ISR. Because of the absence of mesorectum around the very low rectum, the risk of metastases in the mesorectum under the tumor is nil (32). So, the expected benefit of a clear DRM is only for tumor resection and not for mesorectal excision.

For years, it has been considered that a DRM ≥ 5 cm was necessary to optimize the oncological result (33). This 5 cm cut-off did not permit ISR for tumors of the very low rectum for which an APR was the only option.

Recent studies have found that a DRM of 2 cm (34) and also 1 cm does not alter the oncological prognosis with (35) or without preoperative radiotherapy (36). To go even further, some studies question the value of a distal margin of 1 cm (37, 38).

However, these results must be considered with great caution. Some studies focused on non-fixed specimens while others looked at fixed specimens (39). This technical detail is essential since a study dedicated to this question revealed on 26 specimens of colonic and rectal resection that 12–18 h after the fixation in formaldehyde the DRM was reduced by 57% compared to the DRM measured before fixation (40).

Interestingly, by considering the DRM as a continuous variable, Nash et al. reported that the extent of the DRM is significantly associated with disease-free survival in multivariate analysis independently of pre-operative radiotherapy (41). The relevant conclusion of this work relates the importance of guaranteeing a safety DRM in rectal surgery without being able to determine a precise cut-off. A Norwegian registry study involving 3,571 patients after proctectomy for rectal cancer found a negative impact on tumor recurrence when the DRM was <10 mm (36). It is therefore reasonable to aim for a DRM ≥ 1 cm below the tumor when considering ISR. Since after fixation, this margin will decrease by more than 50%, the objective of a margin of 1 cm for the surgeon will guarantee a margin ≥ 5 mm after fixation of the specimen. Interestingly, neoadjuvant treatments do not modify this cut-off (42).

This DRM of 1 cm leads on the one hand to propose an ISR for tumors located at <1 cm from the anorectal ring and on the other hand to incise 1 cm under the tumor during surgical resection. Noteworthy, the DRM is not a criterion for excluding patients for ISR since this surgical technique allows the achievement a safe DRM in any tumor location as suggested by Rullier et al. (43).

As an overview of the resection margins, a CRM > 1 mm and a DRM ≥ 1 cm constitute the objectives. Since the resection cannot be laterally extended to the external sphincter (except in exceptional cases), invasion of the intersphincteric plane is a contraindication to ISR because CRM ≥ 1 mm cannot be performed. At the opposite, DRM is rarely a contraindication to surgery since it is almost always possible to incise 1 cm below the lesion during ISR.

The benefit of neoadjuvant treatments with radiotherapy or radiochemotherapy in lesions of very low rectum to increase the sphincter preservation rate is controversial in the literature. As reported in the review of the literature of Shirouzu et al. the rate of radiochemotherapy before ISR varied widely between each surgical team from 0 to 100% (44). It has not been possible to establish an increase in the chances of sphincter preservation thanks to neoadjuvant treatments (45–47). Nevertheless, these studies have often confused all rectal cancers without specifically considering very low rectal cancers. In addition, the doses of radiotherapy, whether or not combined with chemotherapy, and the delay between radiotherapy and surgery were variable. All these elements clearly biased the evaluation of radiotherapy in this context. In addition to the benefit of radiotherapy in rectal cancer on the survival when indicated (48), this treatment allows downstaging, downsizing of the lesion with 56% of patients with tumor regression grading 3–4 (49) and reducing tumor deposit, budding, and micrometastasis (50). Thus, when the CRM predicted on imaging is under 1 mm, neoadjuvant radiochemotherapy is recommended to optimize the chances of oncologic survival with preservation of the sphincter (51).

In a systematic review of outcomes after ISR for low rectal cancer, summarizing 14 studies including 1,289 patients, overall 5-year survival was 86% and disease-free 5-year survival was 79%. R0 resection was achieved in 97% of cases (52). In this systematic review, the administration of preoperative radiotherapy was highly variable between each study analyzed (from 0 to 100%). Of course, it is difficult to compare survival after ISR with that of APR since ISR has been developed without any prospective randomized controlled trial. To control this evident bias of selection, a retrospective study used a propensity score matching to compare 112 patients after ISR to 173 patients after APR for very low rectal cancer without preoperative therapy (25). After propensity score matching, 5-year relapse-free survival rates were 69.9% for the ISR group and 67.9% for APR group (*p* = 0.64). Similarly, the 3-year cumulative local recurrence rate did not differ between the two groups (7.3% in ISR group vs. 3.9% in APR group, *p* = 0.10). A recent study assessed the factors altering the disease-free survival after ISR in a cohort of 147 patients after preoperative chemoradiotherapy (28). On multivariate analysis, ypT stage, ymrT stage, and circumferential resection margin status were associated with worse disease-free survival.

To summarize, ISR is indicated for rectal tumor <1 cm from the anorectal ring for which a CRM ≥ 1 mm can be guaranteed. Invasion of the external sphincter is therefore a contraindication.

## Functional Approach

To obtain an overview of the anal function after an ISR, Martin et al. in their review of the literature found on average 2.7 bowel movements per 24 h, 51.2% of patients had a perfect continence, 29.1% of patients reported fecal soiling, 23.8% an incontinence to flatus, 18.6% an urgentury, and 18.4% took antidiarrheal medications (52). With a long-term follow-up, this functional results trend to improve over time (53).

The aim of the ISR as an ultimate strategy of sphincteric conservation is to avoid permanent colostomy. The benefit of avoiding permanent colostomy on the quality of life is controversial. A statistically higher quality of life thanks to sphincteric conservation has already been found (54), but this result was not confirmed by the meta-analysis from the Cochrane (55). This situation of sphincteric conservation by ISR exposes the patient to both the consequences of conventional proctectomy as the low anterior resection syndrome or urogenital lesions and to specific continence dysfunction because of the internal sphincter resection. Kupsch et al. have evaluated that 73% of patients described a low anterior rectal syndrome after ISR instead of 58% of patients after total mesorectal excision and 38% of patients after partial mesorectal excision (56). Bretagnol et al. disclosed a significant alteration of the continence in patients after ISR (Wexner score 10.8) vs. patients after conventional coloanal anastomosis (Wexner score 6.9) (*p* < 0.001), and an increase in antidiarrheal medication (57). But in this study the global quality of life was similar between these two groups. A more recent study observed the same comparison with a worse continence after ISR vs. conventional coloanal anastomosis evaluated by the Wexner Score but not reduction in the quality of life (58). Moreover, the risk of definitive stoma 10 years after sphincter-saving resection, for rectal cancer was 18% after partial ISR and 19% after total ISR and these rates did not differ from the risk of a definitive stoma after a conventional coloanal anastomosis (18%) (*p* = 0.578) (59). In this study, the two independent risk factors for definitive stoma were age >65 years and surgical morbidity. Regarding specifically the consequences of surgical morbidity on the functional results, Yokota et al. showed that a major anastomotic leakage impact temporary the anal function that recovered over 2 years only in absence of anastomotic dehiscence (60). In this study, the development of an anastomotic dehiscence affected the functional result over after 2 years. In another work, anastomotic stricture as a delayed surgical complication impacted negatively the anal function (61).

Pre-operative radiochemotherapy represents an independent risk factor of continence dysfunction after ISR (61) and is associated with a lower colostomy-free survival (62).

The impact of the extent of sphincter resection on the functional result is controversial in the literature. Ito et al. did not find any association between the extent of excision of the internal sphincter and an alteration of the functional function (61). Even more surprisingly, Saito et al. did not observe an aggravation of the continence or of the quality of life between internal sphincter resection alone vs. internal sphincter resection with partial resection of external sphincter (63). A multicentric Japanese study did not report the same results as in this study analyzing 228 patients who underwent an ISR, patients with total intersphincteric resection displayed significantly worse continence than patients with partial or subtotal resection (9). In the same way, Denost et al. searched for risk factors for fecal incontinence after ISR and in the results of the multivariate analysis, the only independent predictors of good continence were distance of the tumor >1 cm from the anorectal ring (OR, 5.88; 95%CI [1.75–19.80]; *p* = 0.004) and anastomosis higher than 2 cm above the anal verge (OR, 6.59; 95%CI [1.12–38.67]; *p* = 0.037) (53). These two factors are clearly correlated to the extent of the sphincteric resection.

Different types of anastomosis such as colonic J-Pouch have been evaluated in order to improve the functional results after a proctectomy (64). A recent randomized controlled trial did not find the superiority of a reservoir confection by colonic J-Pouch or side-to-end anastomosis and straight anastomosis on the anal function (65). This controversial issue was not evaluated in the specific setting of ISR but in conventional coloanal anastomosis. A reservoir is always performed when possible to maximize the chances of good functional result without increasing the risk of anastomotic leakage (66).

The last important point to be discussed is the impact of the pre-operative anal function that can be evaluated by anorectal manometry. This exam helps to understand the mechanisms of the continence troubles affected by ISR. The Maximum Resting Pressure (MPR) measured by anorectal manometry is mainly assured at 55% by the internal sphincter anal, although the external sphincter and the hemorrhoidal plexus contribute, respectively, at 30 and 15% for the MPR (67). So that, in a study assessing the performance of anorectal manometry before ISR in 68 patients, a high MPR (>60 mmHg) before surgery was independently associated with fecal incontinence after resection of the internal sphincter (68). In addition, a high-pressure zone ≤ 3 cm measured by preoperative high-resolution anorectal manometry was the second risk factor of fecal incontinence after multivariable analysis. Interestingly, in comparison to subtotal/total ISR, patients after partial ISR had lower risk of fecal incontinence and had significantly a higher MPR and a higher maximum squeeze pressure post-operatively. Other exams have been evaluated as dynamic MRI. As this exam is poorly correlated to patient-reported symptom severity (69, 70), its place is restricted to patients with an externalized or suspected prolapse.

## Patient Selection

Patient selection for ISR requires simultaneous consideration of the oncology and functional approaches outlined above. In summary, the ISR concerns lesions of the very low rectum located <1 cm from the anorectal ring (type II) as well as lesions invading the internal sphincter but preserving the intersphincteric plane and the external sphincter (type III) (8). The choice between a partial, a subtotal or a total resection of the internal sphincter depends on the localization of the inferior edge of the tumor as a DRM of 1 cm should be obtained (71).

Some teams have considered the ISR with a partial resection of the external sphincter for tumors invading the external sphincter (type IV) (9, 72). In our point of view, the oncological safety is uncertain in this situation as the literature is very limited, restricted to few teams. The functional result is largely compromised by this extensive resection. So, in our center, the invasion of the external sphincter persistent after neoadjuvant radiochemotherapy is still an indication for an APR.

Many studies have explored the risk factors of a bad functional result after ISR underlining the importance of the preoperative function (9, 57–63, 68, 72). But, many different factors have been identified and differed significantly between all of these studies. So that, it is extremely difficult to predict the functional result efficiently when ISR is planned. Pre-operative anorectal manometry might bring some help but is not enough to be certain of the post-operative anal function (68). This exam is needed only if there is any doubt about a poor post-operative functional result. In addition, the quality of life is not perfectly correlated to the functional results (58). Thus, the predicted functional result must be cautiously evaluated before an ISR and though about with the patient. As many solutions exist when the anal function is altered, the decision of contraindicate an ISR only because of the predicted functional results should be well-considered. In daily practice, we avoid ISR only in patients with reported incontinence episodes. Denost et al. reported that with a step-up strategy face to a bad anal function with low fiber diet, bulking agents, glycerol-based enemas, loperamide, sphincter re-education by biofeedback, sacral nerve stimulation, and cecostomy for anterograde enema, only 5% of the patients need a definitive stoma formation due to major and refractory fecal incontinence in their experience (73).

Pre-operative radiotherapy deters the anal function (61) but allows downstaging and downsizing of the lesion (49) increasing the chances of a margin-free resection. In case of tumor invading the internal sphincter with a CRM <1 mm, pre-operative radiochemotherapy is clearly justified as the functional considerations should not be deleterious to oncological considerations. Face to a less invasive tumors without an important invasion of the internal sphincter, the best strategy is more difficult as the it can be performed without neoadjuvant radiochemotherapy to improve the functional result but a local excision without ISR can be proposed after an efficient radiochemotherapy (74, 75). This dilemma applies only for tumors within the GRECCAR-2 criteria (i.e., tumor size <4 cm, mrT2–T3) (74). Advantages and drawbacks have to be evaluated in an experienced team and the preference of the patient should be integrated to the final decision. If a non-conservative approach is preferred for these T1/T2N0 lesions, no preoperative radiochemotherapy is required.

## Surgery

After a systematic bowel preparation (76), the surgical procedure can be started by abdominal approach or perineal approach first. As demonstrated by Kanso et al. a perineal approach first reduces operative time without increasing or reducing surgical morbidity, CRM, DRM overall and disease-free survival (77). Of course, with perineal approach first, exploration of the peritoneal cavity to find a peritoneal carcinomatosis is not possible. However, the risk of peritoneal carcinomatosis is rare in very low rectal cancer and is more infrequent than in upper rectal cancer (78). An important technical point to be considered is when the perineal dissection should be stopped. According to retrospective studies synthesized in a recent meta-analysis, transanal total mesorectal excision in comparison to laparoscopic total mesorectal excision significantly improved overall morbidity (34 vs. 41%, OR = 0.65, 95%CI 0.46–0.91, *p* = 0.001), major morbidity (8.7 vs. 14%, OR = 0.53, 95%CI 0.34–0.83, *p* = 0.005), anastomotic leak (6.4 vs. 11.6%, OR = 0.53, 95%CI 0.31–0.93, *p* = 0.03), and circumferential resection margin involvement (4 vs. 8.8%, OR = 0.48, 95%CI 0.27–0.86, *p* = 0.01) (79). Nevertheless, a recent analysis of the Norwegian national registry reveled an unexpected high rate of local recurrence after transanal total mesorecal excision (80). Because of this alarming result, the Norwegian Col-orectal Cancer Group recommended a temporary halt to the performance of transanal total mesorecal excision, and the Norwegian health authorities declared a national moratorium for this surgical approach of the rectal cancer until the national audit on is complete. Two prospective randomized controlled trials are ongoing (81, 82) and their results are indispensable to evaluate the oncological safety of the transanal total mesorectal excision and so to define when the perineal dissection should be stopped during perineal approach first for an ISR.

An important step that should not be forget is the insertion in the canal anal of a gauze with tumoricidal solution (betadine) and the closure of the distal rectum as soon as possible to reduce the risk of tumor-cell dissemination (71).

After resection of the surgical specimen, a handsewn coloanal anastomosis is made with a reservoir to maximize the chances of good functional result without increasing the risk of anastomotic leakage (83). In addition, a diverting loop is systematically performed.

The benefit of a systematic drainage after an ISR has not been specifically assessed but the GRECCAR-5 trial found that the systematic use of a pelvic drain after rectal excision for rectal cancer did not confer any benefit to the patient (84). In our view, a systematic drainage is not justified after an ISR as in this situation the anastomosis in lower than other proctectomies so distant from an intra-abdominal drain.

## Conclusion

Intersphincteric resection, when indicated, offers an interesting alternative to definitive terminal colostomy in case of very low rectal cancer. Thanks to a careful patient selection, this strategy is not just a compromise between quality of life and cancer treatment but represents an optimal oncological treatment for very low rectal cancer. Intersphincteric resection is a model of what surgery is expected to be in the twenty-first century: more and more effective on the disease, less and less damaging for the patient.

## Author Contributions

MC and JL writing, correction, and submission of the manuscript.

### Conflict of Interest

The authors declare that the research was conducted in the absence of any commercial or financial relationships that could be construed as a potential conflict of interest.
